# Tongue Leptin Decreases Oro-Sensory Perception of Dietary Fatty Acids

**DOI:** 10.3390/nu14010197

**Published:** 2021-12-31

**Authors:** Hameed Ullah, Amira Sayed Khan, Babar Murtaza, Aziz Hichami, Naim Akhtar Khan

**Affiliations:** Nutritional Physiology & Toxicology Team, INSERM UMR 1231, Faculté des Sciences de la Vie, Université de Bourgogne-Franche Comté (UBFC), 6 Boulevard Gabriel, 21000 Dijon, France; hameed_ullah@etu.u-bourgogne.fr (H.U.); amira.khan@u-bourgogne.fr (A.S.K.); babarmurtaza87@gmail.com (B.M.); aziz.hichami@u-bourgogne.fr (A.H.)

**Keywords:** fat taste, lipids, fatty acids, obesity

## Abstract

Leptin, an anorectic hormone, regulates food intake, energy expenditure and body weight. We assessed the implication of tongue leptin in the modulation of oro-sensory detection of dietary fatty acids in mice. The RT-PCR analysis showed that mRNA encoding leptin and leptin receptor (Ob-Rb) was expressed in mice taste bud cells (TBC). Confocal microscopic studies showed that the lipid sensor CD36 was co-expressed with leptin in mice TBC. Silencing of leptin or Ob-Rb mRNA in tongue papillae upregulated preference for a long-chain fatty acid (LCFA), i.e., linoleic acid (LA), in a two-bottle paradigm in mice. Furthermore, tongue leptin application decreased the preference for the LCFA. These results suggest that tongue leptin exerts an inhibitory action on fatty acid preference. In isolated mice TBC, leptin decreased LCFA-induced increases in free intracellular calcium concentrations, [Ca^2+^]i. Leptin and LCFA induced the phosphorylation of ERK1/2 and STAT-3 and there were no additive or opposite effects of the two agents on the degree of phosphorylation. However, leptin, but not the LCFA, induced phosphoinositide-3-kinase (PI-3-K)-dependent Akt phosphorylation in TBC. Furthermore, leptin induced hyperpolarization, whereas LCFA induced depolarization in TBC. Our study demonstrates that tongue leptin exerts an inhibitory action on oro-sensory detection of a dietary fatty acid by interfering with Ca^2+^ signaling and membrane potential in mice TBC.

## 1. Introduction

Leptin, the product of the *ob* (obese) gene, is produced by the adipose tissues and other organs [1,2]. Leptin promotes weight loss, by stimulating the rate of metabolism and suppressing appetite. Leptin also signals nutritional status to several other physiological systems and modulates their functions [1]. The defect in the *ob* gene contributes to suppressed leptin production and, ultimately, causes diabetes and development of severe obesity [3]. 

The sense of taste informs the organism about the quality of ingested food. There are, so far, five identified basic taste qualities, i.e., sweet, sour, bitter, salty and umami. Recent compelling evidence from rodent and human studies raises the possibility of an additional sixth taste quality, devoted to the perception of dietary fat. Two principal glycoproteins, CD36 and GPR120, are involved in fat taste perception [4]. The CD36 is a scavenger receptor, whereas GPR120 is a G-protein coupled receptor (GPCR) and belongs to the seven transmembrane domain receptors family [5]. The implication of CD36 in the gustatory perception of fat was shown by employing the double-choice test. The experiments on wild type and CD36 knock-out mice demonstrated that the latter animals completely failed to exhibit a preference for dietary fatty acids [6]. We have shown that CD36 and GPR120 act in a synergic and complementary fashion to control fat eating behaviour [7,8].

Several factors have been shown to influence fat intake and the control of eating behaviour. The hormones/peptides released by the gut during the post-prandial phase of food intake trigger satiation. Agents such as glucagon-like peptide-1 (GLP-1), cholecystokinin (CCK), neuropeptide-Y (NPY), pancreatic polypeptide, and ghrelin modulate food ingestion. Leptin and insulin signal adiposity in both short and long-term energy balance homeostasis [9]. It is interesting to mention that these gut-derived peptides/hormones have a short half-life, and rapidly degrade during food ingestion. Hence, several recent studies have demonstrated that taste buds also release a number of these peptides/hormones including leptin and CCK, and control the oro-cephalic phase of food intake [10]. Leptin is a very interesting hormone as it is released not only by the epithelial cells of the stomach but also by lingual papillae in the vicinity of taste bud cells [11]. However, no study is available on the role of leptin, particularly that released by taste papillae, in the modulation of dietary fat intake. In this study, we assess the implication of tongue leptin in the oro-sensory perception of a dietary long-chain fatty acid (LCFA) in mice.

## 2. Materials and Methods

### 2.1. Animals

The C57BL/6J, 8 weeks old, male mice were purchased from Janvier lab (Le Mans, France). They were kept in the animal house at a constant temperature (25 °C) and humidity (60 ± 5%) with a 12-h light/dark cycle. Body weight, food intake, and water drinking were monitored weekly. The mice were fed ad libitum with standard laboratory chow. The study was conducted as per the Declaration of Helsinki and European ethical guidelines for the care and use of animals for experimentation. The Regional Ethical Committee of the University of Burgundy (Dijon, France) approved the experimental protocol.

### 2.2. Chemicals

All the chemicals, unless specified, including xanthan gum, linoleic acid and anti-mouse secondary antibody, were purchased from Sigma (St. Quentin Fallavier, France). Fura-2/AM was purchased from Life Technologies (Bordeaux, France). Trypsin, RPMI 1640 medium and fetal calf serum (FCS) were bought from Lonza Verviers (Belgium). Pegylated mouse leptin antagonist (mutant L39A/D40A/F41A) was purchased from CliniSciences (Montluçon, France). Accell siRNA for leptin, leptin receptor (Ob-Rb) and non-targeting (labeled with DY-547) were procured from Dharmacon™ (USA). The ELISA kit for leptin (A05176) was obtained from Bertin Technologies (Aix-en-Provence, France). Alexa 568 donkey anti-rabbit (A10042), Alexa 488 green goat anti-rabbit (A11034) and other molecular biology reagents including TRIzol were from Invitrogen (Waltham, MA, USA). The rabbit anti-CD36 (HPA002018) antibody was bought from Atlas (Cambridge, UK). The anti-β-actin antibodies were from Santa Cruz Biotechnology (Dallas, Texas, USA). The anti-leptin (GTX17629) and anti-Ob-Rb (GTX27211) antibodies were bought from GeneTex (Irvine, CA, USA). The anti-rabbit (7074S) goat antibodies were bought from Cell signaling (Charles-Renard, France). The polyvinylidene fluoride (PVDF) membranes and enhanced chemiluminescence (ECL) substrate were procured from Bio-Rad (Marnes-la-Coquette, France).

### 2.3. Isolation of Taste Bud Cells (TBC)

Mice were anesthetized by sedation in a CO_2_ chamber and sacrificed as reported previously [12]. The CD36-positive taste bud cells (TBC) from tongue circumvallate papillae were isolated by an enzymatic (elastase/dispase) mixture in Tyrode buffer (120 mM NaCl, 5 mM KCl, 10 mM HEPES, 1 mM CaCl_2_, 10 mM glucose, 1 mM MgCl_2_, 10 mM Na pyruvate, pH 7.4), as described before [12]. Isolated TBC were used immediately or stored at −80 °C for further analysis.

### 2.4. siRNA Application onto the Mouse Tongue and Detection of Silenced Targets

The siRNA against leptin, leptin receptor (Ob-Rb) or control (non-targeting) were processed as per manufacturer’s instructions. The three tongue papillae (circumvallate, fungiform and foliate) were targeted by siRNA. Each mouse was anesthetized with isoflurane (1.5–3% in oxygen) and 5 μL of siRNA was slowly pipetted onto the tongue (covering ¼ caudal and ¾ rostral regions) as reported previously [13]. This process was repeated for 4 consecutive days. Hereafter, the mice were subjected to either of the protocols (two-bottle choice test, TBC isolation for confocal microscopy, or RT-PCR analysis).

For confocal localization of non-targeting siRNA (labeled with DY-547), the freshly isolated TBC were transferred onto glass poly-L-lysine coated coverslips, then fixed with paraformaldehyde, PFA (4%, *v*/*v*), washed with PBS, mounted with ProLong™ diamond antifade mountant with DAPI and subjected to microscopic observations, using a 40 × 1.25 or 63 × 1.4 oil immersion objective lens in sequential mode. The microscope was equipped with a laser beam (absorbance/emission filters were 548/562 nm) and the emitted fluorescence was recorded using a PMT or Hybrid detector. The images were acquired by LAS X software (Leica Microsystems, Wetzlar, Germany) at a resolution of 1024 × 1024 pixels, and a scan speed of 600 Hz. To assess the thickness of the structure and obtain 3D views, a series of optical sections at 0.3 µm intervals in the z-axis was taken.

To assess whether two siRNA, encoding leptin and Ob-Rb, decrease the expression of their respective targets in gustatory cells, immunocytochemical localization was performed. Hence, the TBC, cultured for 24 h, were washed with PBS and further centrifuged at cytospin (1500 rpm × 5 min). Cells were fixed with PFA (*v*/*v*, 4%) for 10 min, permeabilized with 0.5% (*w*/*v*) Triton X-100 in PBS and saturated with PBS-BSA (*v/w*, 3%). The slides were incubated with rabbit anti-leptin or anti-Ob-Rb antibody (1/200 dilution in PBS, BSA 1%, *w*/*v*). Slides were washed with PBS three times and then incubated with secondary antibodies, alexa-488 goat anti-rabbit (1:500 dilution in PBS, BSA 1%, *w*/*v*) for 45 min. Staining specificity was assessed by carrying out the same procedure but omitting the primary antibodies. Then, slides were washed three times with PBS and 1 drop of mounting media (ProLong™ Diamond antifade mountant with DAPI) was added onto the slide. The cells were observed under a fluorescent microscope (Zeiss Axioskop, Rueil Malmaison, France).

### 2.5. Confocal Microscopy of Co-Localization of Leptin, Ob-Rb with CD36

Confocal microscopy was used for the colocalization of leptin and leptin receptor (Ob-Rb) with CD36. The freshly isolated TBC were immediately transferred onto the poly-L-lysine coated slides and, after fixation, permeabilization and saturation as described before, slides were incubated overnight with either rabbit anti-leptin or anti-Ob-Rb antibodies diluted in PBS-BSA (*v/w*, 1%). After washing with PBS, slides were incubated with secondary antibodies (green florescent alexa-488 goat anti-rabbit antibody). The same slides were incubated for a second round for 6h with rabbit anti-CD36 antibodies (dilution 1/200), followed by incubation with secondary antibodies (red florescent-568 anti-mouse; 1:500 dilution in PBS, BSA 1%) for 45 min. After washing, the slides were dried and mounted in mounting media with DAPI then observed in the confocal microscope as described above.

### 2.6. Two-Bottle Preference Test

Mice were applied orally with either leptin or PBS at a dose of 24 µg/100 µL/day/mouse for 8 continuous days. In another set of experiments siRNA was applied on tongue as described above. At the end of these treatments, the experiments on the spontaneous preference for fatty acid-containing solutions were performed by a 2-bottle preference test as described elsewhere [14]. Mice were deprived of water for 6 h and further subjected to two bottles: one containing a fatty acid (linoleic acid at 7.1 mM, dissolved in xanthan gum (0.3%, *w*/*v*) and other containing control solution (xanthan gum 0.3%, *w*/*v*) for 12 h. The mice had to choose between a control solution and a fatty acid solution. The intake was determined by weighing the water bottles before and at the end of 12 h period.

### 2.7. Western Blot Analyses

The mice TBC (1 × 10^6^ cells/assay) were lysed by a buffer that consisted of 20 mM HEPES, pH 7.3; 1 mM EDTA; 1 mM EGTA; 0.15 mM NaCl; 1% Triton X-100; 10% glycerol; 1 mM phenylmethylsulphonyl fluoride (PMSF); 2 mM sodium orthovanadate and 2 μL/mL anti-protease cocktail. The lysed samples were kept on ice for a period of 30 min and then centrifuged (12,000× *g* 10 min, 4 °C). The lysates were immediately used or stored at −80 °C until the assay. The protein concentrations in the samples were assayed by using the BCA Kit from Sigma (Saint-Quentin-Fallavier, France). The protein samples (25 μg/well) were separated on SDS-PAGE (8% and 12% for Ob-Rb and leptin, respectively). Separated proteins were transferred to PVDF membranes and later blocked for three hours by adding the TBS buffer that contained BSA (5%, *w*/*v*) and Tween-20 (0.5%, *v*/*v*). The PVDF membrane was probed at 4 °C overnight with primary anti-leptin (1/200, *v*/*v* dilution) and anti-Ob-Rb antibodies (1:1000, *v*/*v*), followed by a thorough washing and incubation at room temperature for 2 h with peroxidase-conjugated secondary antibody (1:2000, *v*/*v*). The detection of blots was performed by chemiluminescence (Bio-Rad, Marnes-la-Coquette, France).

### 2.8. ELISA Detection of Secreted Leptin

The secretion of leptin in the culture supernatants of TBC was assessed by using sandwich enzyme-linked immunosorbent assay (ELISA). The microtiter plates were pre-coated with anti-leptin antibodies. Standards or samples were added to the appropriate ELISA plate wells. Then, a biotinylated antibody and avidin-horseradish peroxidase (HRP) conjugates were added to each well and incubated. The enzyme-substrate reaction was terminated by the addition of sulfuric acid. The optical density (OD) was measured spectrophotometrically at a wavelength of 450 nm.

### 2.9. Calcium Signaling

The increases in free intracellular calcium concentrations, [Ca^2+^]i, were determined as described elsewhere [14]. Briefly, TBC (1 × 10^6^ cells/well) were seeded onto Willico-Dish wells and incubated with Fura-2/AM at 1 µM for 30 min in the buffer (110 mM, NaCl; 5.5 mM, KCl; 25 mM, NaHCO_3_; 0.8 mM, MgCl_2_; 0.4 mM, KH_2_PO_4_; 0.33 mM, Na_2_HPO_4_; 20 mM, HEPES; 1.2 mM, CaCl_2_) with a pH 7.4. The changes in [Ca^2+^]i were monitored using a Nikon microscope (TiU) equipped with EM-CCD (Luca-S) camera with S-fluor 40× immersion oil objective (Nikon, Tokyo, Japan). Changes in [Ca^2+^]i were expressed as F_340_/F_380_ ratio. All test molecules were added in small volumes with no interruptions in recordings.

### 2.10. RT-qPCR

Total RNA from TBC was extracted by using Trizol (Invitrogen Life Technologies, Groningen, The Netherlands) and further subjected to DNAse treatment (Qiagen, Germany). One μg of total RNA was reverse transcribed with Super script II RNAse H-reverse transcriptase using oligo (dT). RT-PCR was performed on iCycler iQ real time detection system by using SYBR Green I. The amplification reaction contained 5 μL SYBR^®^ Green super-mix buffer [50 mM, KCl; 20 mM, Tris–HCl (pH 8.4); 3 mM, MgCl_2_; 0.2 mM of each dNTPs, 0.63U iTaq DNA polymerase and SYBR^®^Green 1.0 nM fluorescein] and 0.5 μL (0.3 μM) of each primer and diluted cDNA. Results were evaluated by iCycler iQ software. The relative gene expression was determined using ΔΔCt method. The normalized delta cycle threshold (ΔCt) was calculated by subtracting the genes of interest cycle threshold value from the β-actin cycle threshold value (ΔCt = Ctβ-actin-Ct gene). Comparative gene expression between two independent samples, or ΔΔCt, was determined by subtracting the vehicle delta cycle threshold from the sample delta cycle threshold (ΔΔCt = ΔCt sample-ΔCt control). Fold change expression was defined with 2^-(ΔΔCt)^. The sequences for the primers used in this study are shown in Table 1.

### 2.11. Measurement of Plasma Membrane Potential

The plasma membrane potential was determined by using the dye, bis-oxonol, as mentioned elsewhere [13]. The TBC were prepared as for the calcium signaling experiments. After washing, the cells (1 × 10^6^ cells/assay) were transferred to the fluorometer cuvettes and 150 nm of bis-oxonol was added. The TBC were allowed to equilibrate with the dye and, after 10 min, different test molecules were added. The fluorescent intensities were determined at 540 nm (excitation filter) and 580 nm (emission filter). Upward deflections represent depolarizations whereas downward responses represent hyperpolarization.

### 2.12. Statistical Analyses

Results are shown as mean ± SEM (standard error of the mean) for a given number of experiments (*n*). Data were analyzed by using Statistica software (4.1 version, Statsoft, Paris, France). The significance of differences between mean values was determined by two-tailed students *t*-test. Differences with *p* < 0.05 were considered significant.

## 3. Results

### 3.1. Mouse TBC Express Leptin, Ob-Rb and CD36

**Figure 1:**
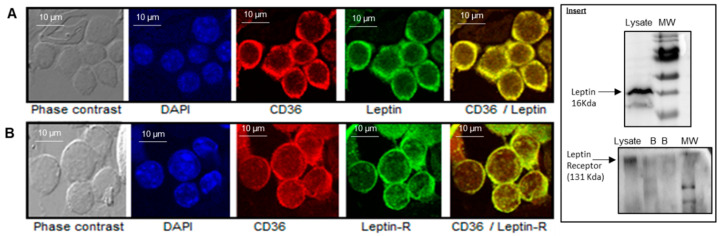
Co-expression of lipid sensor CD36 with leptin and leptin receptor (leptin-R) in mouse TBC. (**A**,**B**) show images acquired with Leica TCS-SP2 confocal laser scanning microscope. Immunoreactivity for CD36 (red) and leptin receptor (green) was observed in cultured TBC. Nuclei of cells were stained with DAPI (**A**,**B**). Insert shows western blot of leptin and leptin receptor. The TBC (1 × 10^6^ cells per assay) were lysed and processed for western blotting as described in Materials and Methods. B (insert) = blank without protein. MW = molecular weight.

Figure 1 shows that CD36 is expressed in freshly isolated mice TBC. Leptin and Ob-Rb expression is also visible in mice TBC (Figure 1A,B). The merged photographs of CD36 with leptin and Ob-Rb clearly show co-expression of lipid sensor with this anorectic hormone and its receptor.

We are interested in assessing the expression of leptin receptor (Ob-Rb) at protein level. Figure 1 Insert shows that mouse TBC express leptin receptor (Ob-Rb) as well as leptin.

### 3.2. Silencing of Leptin and Ob-Rb Genes Increases Gustatory Perception for Dietary Fatty Acid

In order to demonstrate the implication of leptin and Ob-Rb receptor in the modulation of preference for dietary lipids, we employed the siRNA technology.

**Figure 2:**
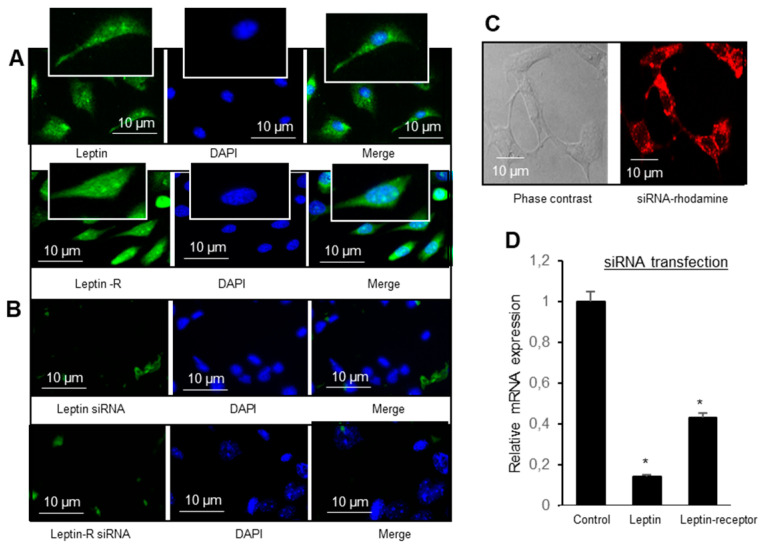
Expression of leptin and leptin receptor (Ob-Rb) in mice TBC. (**A**) Immunostaining of leptin and leptin receptor, leptin-R, (green in (**A**)), in cultured TBC, were acquired through fluorescent microscope, nuclei of the cells were stained with DAPI. (**B**) shows the absence immunostaining of leptin and leptin-R in TBC after their siRNA application onto mice tongue. (**C**) shows the incorporation of non-targeting siRNA (labeled with DY-547) by mouse TBC. (**D**) shows leptin and leptin-R mRNA expression after application onto the tongue of their respective siRNA. The asterisks in D show significant values (*p* < 0.001) compared to control group.

At first hand, we wanted to be sure that exogenous siRNA is incorporated into mouse TBC. Figure 2C shows that non-targeting siRNA applied daily for 4 days was incorporated into TBC. Furthermore, Figure 2A shows that leptin and its receptor, Ob-Rb, are expressed immunocytochemically and their respective siRNA decreased their expression, not only in immunocytochemical studies (Figure 2B) but also at the mRNA level (Figure 2D).

**Figure 3:**
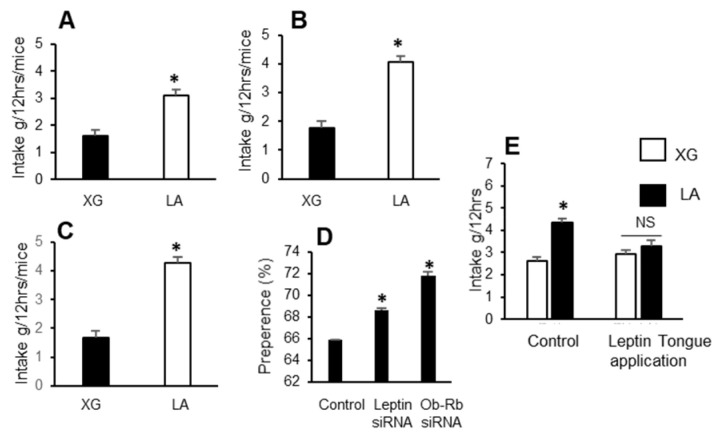
Effect of siRNA on preference for a fatty acid. The control animals (**A**) similarly received the non-targeting siRNA. The siRNA for leptin (**B**) and Ob-Rb (**C**) were applied daily onto the mice tongue for 4 days as described in Materials and Methods. On the 5th day, the mice were subjected to the two-bottle choice paradigm to assess preference for lipids. The mice were deprived of water for 6 h before being subjected to the two-bottle test. One bottle contained the vehicle solution (XG, xanthan gum at 0.3%, *w*/*v*) and another contained linoleic acid (LA) at 0.2% (*w*/*v*) in the xanthan gum (0.3%, *w*/*v*) for 12 h. The histograms in D show the percentage preference, calculated on the basis of results from Figure 3A–C. In Figure 3E, leptin was applied onto mice tongue for 8 consecutive days followed by the two-bottle preference test. The asterisks in A, B and C show significant differences (*p* < 0.001) as compared to control (XG group) animals. In (**D**), asterisks show significant values (*p* < 0.001) compared to control group. In (**E**), asterisk shows the significant difference (*p* < 0.001) in LA group compared to XG group. Abbreviations: LA, linoleic acid; XG, xanthan gum; NS, not significant (differences).

**Figure 4:**
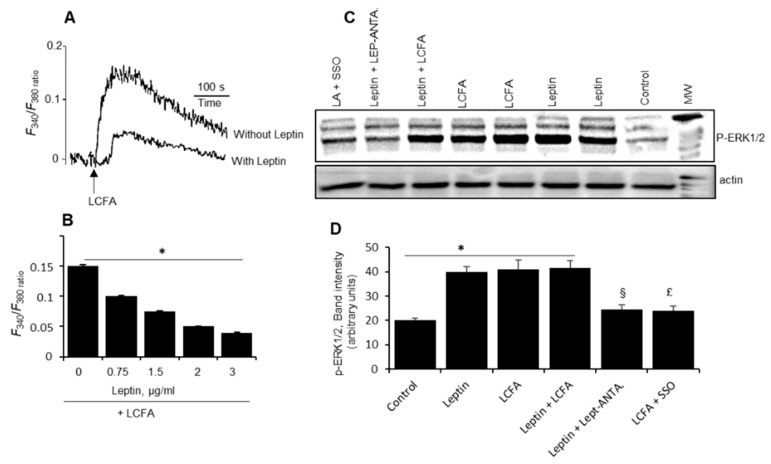
The effect of leptin on Ca^2+^ and MAP kinase signaling. The freshly isolated mice TBC were loaded with Fura-2/AM and resuspended in Ca^2+^- containing medium as described in Materials and Methods. TBC were preincubated or not with leptin (1 µg/mL) for 15 min at room temperature (**A**). The LCFA, i.e., linoleic acid (25 µM), was added into the cuvette without interruptions in the recordings (**A**). The Y-axis represents the Δ increases in [Ca^2+^]_i_. The curves show the single traces of observations which were reproduced several times, independently (*n* = 6). (**B**) shows the increases in [Ca^2+^]_i_, induced by LCFA, in the presence or absence of increasing concentrations of leptin. Asterisk shows the significant differences (*p* < 0.001) compared to LCFA-induced response. (**C**,**D**) show ERK1/2 phosphorylation. The cells were treated with different agents [leptin, 1 µg/mL; LCFA, 25 µM; sulfo-*N*-succinimidyl oleate (SSO), 10 µM; leptin antagonist (LEPT-ANTA), 10 nM] for 20 min with or without inhibitors, and then TBC were lysed and processed for western blotting. Asterisk (in **D**) shows the significant differences (*p* < 0.001) compared with control (untreated cells). § and £ show significant differences (*p* < 0.001), compared with, respectively, cells treated with leptin and LCFA alone. Histograms (in **D**) show the relative band intensity (arbitrary units) of p-ERK1/2 measured by densitometry of protein bands (*n* = 5). Abbreviations: MAP, mitogen activated protein; MW, molecular weight.

In a two-bottle paradigm, the mice exhibited a preference for linoleic acid, a dietary LCFA (Figure 3A). However, the mice that received lingual application of leptin and Ob-Rb siRNA exhibited a higher preference for the LCFA than control animals (Figure 4A–D). These results suggest that the downregulation of leptin and Ob-Rb upregulated preference for the LCFA. Interestingly, local application of leptin onto the tongue decreased gustatory preference for LCFA (Figure 3E).

### 3.3. Leptin Interacts with Cell Signaling Cascade and Induces Hyperpolarization

Since leptin inhibited LCFA oro-sensory detection, we assessed different components of the cell signaling cascade in mice TBC.

**Figure 5:**
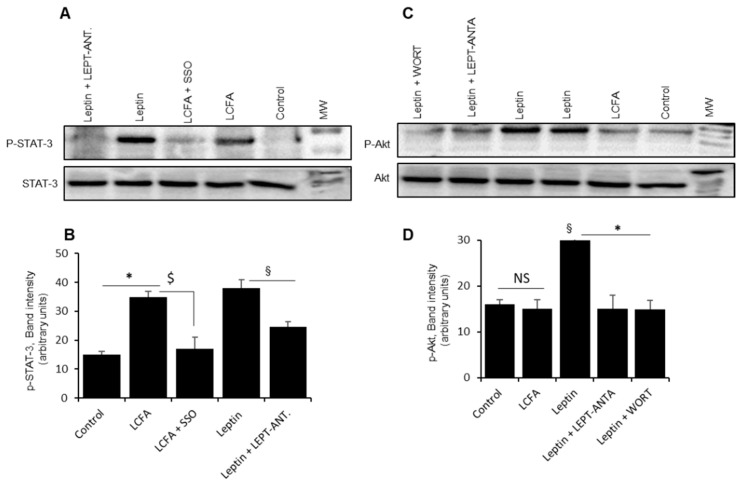
The effect of leptin on STAT-3 and Akt phosphorylation. (**A**) shows phosphorylated and unphosphorylated STAT-3. In (**B**), asterisk shows the significant differences (*p* < 0.001) compared to control (untreated cells), whereas § and $ show significant differences (*p* < 0.001) between two compared groups. (**C**) shows the Akt (phosphorylated and unphosphorylated). The TBC were treated or not (control) with different agents (leptin, 1 µg/mL; LCFA, 25 µM; leptin antagonist (LEPT-ANTA), 10 nM; wortmannin (WORT), 10 nM; sulfo-*N*-succinimidyl oleate (SSO), 10 µM) for 20 min, and then TBC were lysed and processed for western blotting as described in Materials and Methods. In (**D**), asterisk shows the significant differences (*p* < 0.001) compared with leptin treated cells. § shows significant values (*p* < 0.001), compared with control (untreated) cells. NS represents not significant differences. Histograms (**B**,**D**) show the relative band intensity (arbitrary units) measured by densitometry of protein bands. Data were normalized with respect to band intensity of unphosphorylated STAT-3 (**B**) and Akt (**D**), measured under similar conditions (*n* = 5). Abbreviation: MW, molecular weight.

We explored Ca^2+^ signaling, and noticed that LA-induced increases in [Ca^2+^]i were decreased by prior incubation with leptin (Figure 4A,B). Leptin, indeed, decreased Ca^2+^ signaling in a dose dependent manner (Figure 4B). Figure 4C shows that both leptin and LCFA induced the phosphorylation of ERK1/2 in mice TBC. The LCFA-induced ERK1/2 phosphorylation was mediated via CD36 as sulfo-*N*-succinimidyl oleate (SSO), a CD36 blocker, decreased the same. Similarly, the EKR1/2 phosphorylation, induced by leptin, was curtailed by Ob-Rb antagonist. However, both leptin and LCFA did exert an additive effect on ERK1/2 phosphorylation (Figure 4D). Leptin signaling involves STAT-3 phosphorylation. Both LA and leptin triggered STAT-3 phosphorylation and there was no additive effect of both the agents (LCFA and leptin) in mice TBC (Figure 5A,B). Interestingly, SSO and leptin antagonist blocked STAT-3 phosphorylation in mice TBC.

Leptin has been shown to trigger the activation of other signaling pathways, such as activation of phosphatidylinositol-3-phosphate (PI-3P) that induces Akt phosphorylation. To probe the implication of PI-3-P/Akt pathway, we have employed wortmannin that blocks PI-3P. Figure 5C,D shows that leptin, but not LA, induced the phosphorylation of Akt. Both wortmannin and leptin receptor antagonist decreased Leptin-induced Akt phosphorylation in these cells (Figure 5D).

**Figure 6:**
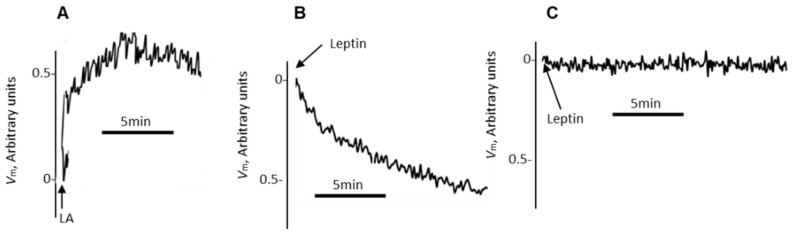
The effects of a LCFA and leptin on membrane potential (*V_m_*) in mouse TBC. The TBC (2 × l0^6^ cells/assay) were loaded with the fluorescent probe, bis-oxonol, as described in Materials and Methods. The arrow heads indicate the time when the test molecules, LCFA, i.e., LA (20 µM) in (**A**) and leptin (10 µg/mL) in (**B**,**C**), were added into the cuvette. The right panel (**C**) shows the action of leptin in TBC, preincubated with wortmannin (100 nM) for 10 min. The Figure shows the single traces of identically reproduced experiments (*n* = 5).

We further assess the changes in plasma membrane potential (*V_m_*) in mice TBC. The Figure 6 shows that LA, the LCFA, induced depolarization in TBC (Figure 6A), whereas leptin induced hyperpolarization in these cells (Figure 6B). The prior-incubation with PI-3-P inhibitor, wortmannin, abolished leptin-induced hyperpolarization in TBC (Figure 6C).

## 4. Discussion

Leptin has been considered as a determinant hormone in modulating eating behavior. This agent mainly inhibits food intake via its action on arcuate nucleus in the brain. That is one of the reasons that leptin deficiency leads to high food intake and, consequently, to obesity. The leptin receptors are expressed not only in the central nervous system but also in the peripheral tissues, including tongue epithelium [11]. It is important to mention that circulating plasma leptin levels have been correlated with alterations in taste thresholds in human beings [15] and rodents [16]. Rodrigues et al. [17] reported that saliva leptin was involved in the modulation of bitter and sweet taste perception in human participants. Hence, our hypothesis is based on these observations that the tongue might be a peripheral target of leptin in modulating fat taste perception.

We observed that mice TBC expressed both leptin and leptin receptor, i.e., Ob-Rb, at the mRNA level, and leptin was co-expressed with lipid sensor (CD36) in these cells. Indeed, mouse fungiform and circumvallate papillae, but not surrounding tongue epithelium, have been shown to express leptin and high-density functional Ob-Rb isoform of leptin receptor [18]. In order to demonstrate the physiological implication of leptin and Ob-Rb in gustatory perception of a dietary fatty acid, we employed siRNA technology to silence their endogenous expression. The siRNA application clearly shows that exogenous siRNA is incorporated into TBC, and silenced the synthesis of leptin and Ob-Rb in these cells. However, silencing leptin and Ob-Rb gene in mice TBC resulted in increased preference for a LCFA in a two-bottle paradigm. Conversely, leptin lingual application decreased the same. These observations clearly demonstrate that leptin exerts an inhibitory action on fat taste perception, as is the case for sweet taste perception [19,20]. Yoshida et al. [21] demonstrated that leptin suppressed sweet taste via Ob-Rb in mice.

How does leptin decrease fat perception, whether in a paracrine or autocrine manner? We assume that it might be an autocrine action, as the TBC-expressing CD36 also express both leptin and Ob-Rb mRNA. We conducted further experiments on the mechanism of leptin’s action on purified mice TBC. An increase in [Ca^2+^]i and phosphorylation of MAP kinase (ERK1/2) cascade belong to the early mechanisms, involved in oro-sensory detection of dietary fat in mice [7,13,22]. We observed that leptin decreased LCFA-induced increases in [Ca^2+^]i in isolated mice TBC. These observations corroborate the findings of Horio et al. [23] who have shown that exogenous leptin decreased sweet-induced calcium signaling in CHO and enteroendocrine STC-1 cells [24]. Interestingly, in CHO biosensor cells, leptin also decreased the release of ATP, an agent that is involved in cell–cell interactions to contribute to neurotransmitter release in TBC [23]. As regards MAPK activation, both LCFA and leptin induced the phosphorylation of ERK1/2 and both agents did not induce an additive effect, demonstrating that MAPK is not involved in the modulatory action of leptin on fat oro-sensory perception in mice TBC.

Ob-Rb was found to be associated with STAT3 at the mRNA level in mice TBC [20]. We observed that both leptin and LCFA triggered the STAT3 phosphorylation in TBC and, when combined, both agents could not influence the state of phosphorylation. Hence, we can exclude the role of STAT-3 in the inhibitory action of leptin on fat taste perception.

It is also possible that there might be other mechanisms involved in the inhibition of fat taste perception. The PI-3-P/Akt is the parallel signaling pathway that is also operated via Ob-Rb in taste bud cells. Interestingly, leptin, but not the LCFA, induced Akt phosphorylation and this phenomenon was reversed by wortmannin, a PI-3-K inhibitor, suggesting that the PI-3-P/Akt pathway might be involved in leptin’s inhibitory action as suggested by Yoshida et al. [20] for sweet taste perception. These authors further demonstrated that leptin-induced PI-3-P/Akt activation was responsible for TBC hyperpolarization which contributed to its inhibitory action on sweet taste perception [20]. Interestingly, we also observed that leptin induced TBC hyperpolarization via the PI-3-P pathway. However, the LCFA induced depolarization in these cells. The TBC depolarization, via the opening of Ca^2+^-activated transient receptor potential melastatin-5 (TRPM5) channels, has been shown to be one of the key events involved in the release of neurotransmitters, responsible for the transmission of the gustatory message towards the brain [25]. Our observations demonstrate that leptin might exert an inhibitory action of gustatory perception of dietary lipids vis its action on two signaling events, i.e., Ca^2+^ signaling and hyperpolarization, in mice TBC.

Finally, we can state that our study contributes to the hypothesis that leptin may regulate fat-rich food intake via its action in the periphery by influencing early mechanisms of fat taste perception in the microenvironment of the taste papillae.

## Figures and Tables

**Table 1:** Sequences of the primers.

Primers	Forward	Reverse
β-actin	TCCTTTGCAGCTCCTTCGTT	ATGGAGGGGAATACAGCCC
Leptin	ACACACGCAGTCGGTATATCC	GAGTAGAGTGAGGCTTCCAGG
Ob-Rb	TGAAAAAGTTGTTTTGGGACG	TGAACACAACAACATAAAGCCC

## Data Availability

The data will be made available upon reasonable request.
